# Microglia Modulate Information Processing in the Mouse Barrel Cortex

**DOI:** 10.1523/JNEUROSCI.0941-25.2026

**Published:** 2026-02-24

**Authors:** Bálint Király, Eszter Császár, Diána Balázsfi, Claire-Hélène de Badts, Katalin Sviatkó, Balázs Pósfai, Andor Domonkos, Balázs Hangya, Ádám Dénes

**Affiliations:** ^1^MTA–HUN-REN KOKI Lendület “Momentum” Laboratory of Systems Neuroscience, HUN-REN Institute of Experimental Medicine, Budapest H-1083, Hungary; ^2^Division of Neurophysiology, Center for Brain Research, Medical University of Vienna, Vienna A-1090, Austria; ^3^“Momentum” Laboratory of Neuroimmunology, HUN-REN Institute of Experimental Medicine, Budapest H-1083, Hungary; ^4^Interdisciplinary Masters’ in Life Sciences, Ecole Normale Supérieure, Paris F-75230, France; ^5^Laboratory of Thalamus Research, HUN-REN Institute of Experimental Medicine, Budapest H-1083, Hungary; ^6^Subcortical Modulation Research Group, HUN-REN Institute of Experimental Medicine, Budapest H-1083, Hungary

**Keywords:** functional connectivity, interneuron, microglia, P2Y12R, sensory processing

## Abstract

Microglia, the main immune cells of the central nervous system, are crucial for maintaining brain homeostasis by modulating immune processes and neurovascular function. However, the mechanisms by which microglia regulate neuronal networks and local microcircuits remain incompletely understood. Here, we identify microglia as important modulators of neuronal network activity at the single-cell level and brain-wide functional connectivity in male mice. We show that in the absence of microglia or microglial P2Y12 receptor (P2Y12R), the baseline firing rate of putative interneurons was increased, while whisker stimulation-induced sensory responses remained unchanged in microglia-depleted and P2Y12R KO animals. Increase in cortical delta oscillations in both models and increased single neuron phase coupling to delta band rhythms in microglia-depleted mice revealed cortical hypersynchrony. Microglia depletion led to a significant reduction in connectivity between the contralateral barrel cortex and the anatomically connected ventral posteromedial nucleus of the thalamus during somatosensory stimulation, while resting-state functional connectivity remained unchanged. Similarly, genetic blockade of P2Y12R resulted in diminished functional connectivity within this thalamocortical network. Our findings suggest that cortical interneuron hyperexcitability due to dysfunction of microglia could be a key cause for local hypersynchrony relevant to sensory processing.

## Significance Statement

Microglia have been shown to modulate neuronal activity, but the underlying mechanisms are insufficiently defined. In particular, it is not well understood how microglia could shape excitatory/inhibitory balance in the cerebral cortex and whether such modulatory processes could alter sensory processing. Here, we studied single-cell–level effects in the barrel cortex by using two established models of microglia dysfunction. We show that the absence of microglia or the purinergic microglial receptor, P2Y12R, have both large-scale effects on thalamocortical networks and cortical slow oscillations while specifically shaping the firing rate of interneurons in cortical microcircuits. Such neuroglial interactions could have broad impact on sensory processing in health and under different disease states.

## Introduction

Microglia, the primary immune cells of the central nervous system (CNS), are essential for maintaining CNS homeostasis and coordinating immune responses to pathogenic challenges (Li and Barres, 2018; Borst et al., 2021). Beyond their immune functions, microglia play crucial roles in brain development and in supporting neuronal and vascular function throughout life (Thion et al., 2018; Zhao et al., 2018; Cserep et al., 2021). Microglia maintain a state of dynamic surveillance in the brain, through which they modulate synaptic integrity, dendritic spine remodeling, and neurogenesis and also shape neuronal activity and network synchronization by interacting with different neuronal compartments (Szalay et al., 2016; Akiyoshi et al., 2018; Badimon et al., 2020; Cserép et al., 2020; Umpierre et al., 2020). Optimal regulation of these processes is fundamental for complex tasks including sensory processing, learning, and memory, while microglia dysfunction is increasingly linked with common neurological diseases, including stroke, Alzheimer's disease, Parkinson's disease, or depression. However, the mechanisms through which microglia contribute to neuronal network regulation and related dynamic neurovascular processes remain incompletely understood.

We and others have recently revealed that microglia are crucial for modulating complex neurovascular processes including the maintenance of cerebral blood flow (CBF) in response to neuronal activity (neurovascular coupling) or hypercapnia-induced vasodilation through P2Y12R-mediated mechanisms (Bisht et al., 2021; Csaszar et al., 2022; Fu et al., 2025). While the absence of microglia or microglial P2Y12R impairs CBF responses to somatosensory stimulation and hypercapnia, we found that altered baseline firing of neurons in the somatosensory cortex in response to microglia manipulation was not associated with altered neuronal responses to somatosensory stimulation (Csaszar et al., 2022). However, individual neuron populations were not investigated separately in previous studies, and hence the impact of microglial actions on the activity of different excitatory and inhibitory neuronal populations is not known. Furthermore, microglial impact on specific information transmission modes such as burst firing (Kepecs et al., 2002; Kepecs and Lisman, 2003), as well as local oscillatory synchrony, has rarely been addressed (Akiyoshi et al., 2018; Berki et al., 2024; Steffens et al., 2024).

Emerging evidence indicates that microglia interact with excitatory and inhibitory neurons in a highly context- and region-dependent manner, which is influenced by subtype-specific electrophysiological activity (Parkhurst et al., 2013; Favuzzi et al., 2021; Ngozi and Bolton, 2022). Impaired microglial function is associated with disruptions in neuronal network function, functional brain connectivity, and behavioral abnormalities through signaling pathways such as complement receptor 3 (CR3), CX3CR1, and TREM2 that are likely to contribute to neurodevelopmental and neuropsychiatric disorders, including autism, schizophrenia, and depression (Paolicelli et al., 2011; Schafer et al., 2012; Zhan et al., 2014; Filipello et al., 2018; Deivasigamani et al., 2023). Our previous work has demonstrated that acute blockade of microglial P2Y12R disrupts cortical functional connectivity following ischemic stroke (Cserép et al., 2020).

However, the effects of microglia dysfunction on sensory-evoked neuronal activity and functional connectivity changes under physiological conditions remain largely unexplored. Specifically, the contribution of microglia to differential regulation of excitatory/inhibitory neurons in local microcircuits and the impact of these actions on brain-wide connectivity changes are not known. To address these questions, we used two complementary models to examine microglial contributions to neuronal activity and functional connectivity. We applied pharmacological microglia depletion by using the CSF1R inhibitor PLX5622 to eliminate microglial modulatory effects across the brain and used P2Y12R knock-out mice to selectively disrupt microglial ADP/ATP signaling via P2Y12R, a key modulator of microglial cell–cell interactions (Davalos et al., 2005; Haynes et al., 2006; Cserép et al., 2020; Csaszar et al., 2022). These two models also enable comparison of global versus pathway-specific effects of microglia–neuron interactions and neuronal responses (Cserep et al., 2021). Using these tools, we investigated whether elimination of microglia or genetic blockade of microglial P2Y12R alters firing properties and stimulus responsiveness of single barrel cortex neurons and whether cortical network oscillations and functional connectivity are altered in mice in response to somatosensory stimulation.

## Materials and Methods

### Animals

Experiments were carried out on 9–14-week-old C57BL/6J (The Jackson Laboratory, catalog #000664) and P2Y12R^−/−^ (B6;129-P2ry12^tm1Dgen^/H P2Y12R KO, Deltagen, catalog #EM:02301) male mice. Electrophysiology experiments were conducted using *n* = 4 control (C57BL/6J), *n* = 4 microglia-depleted (C57BL/6J), and *n* = 5 P2Y12R^−/−^ mice under mild anesthesia. For freely behaving electrophysiology experiments, recordings were obtained from *n* = 7 C57BL/6J mice at baseline and after microglial depletion (following a 3-week PLX5622 diet). For functional ultrasound (fUS) imaging, *n* = 5 control (C57BL/6J) and *n* = 5 microglia-depleted (C57BL/6J) mice were used, and *n* = 9 control (C57BL/6J) and *n* = 9 P2Y12R^−/−^ mice. The experiments were performed according to the regulations of the Hungarian Act of Animal Care and Experimentation (1998; XXVIII, section 243/1998, renewed in 40/2013) in accordance with the European Directive 86/609/CEE and modified according to the Directive 2010/63/EU. Experimental procedures were reviewed and approved by the Animal Welfare Committee of the Institute of Experimental Medicine, Budapest, and by the Committee for Scientific Ethics of Animal Research of the National Food Chain Safety Office of Hungary. Animals were housed individually in 36 × 20 × 15 cm cages under a standard 12 h light/dark cycle (lights on at 8 A.M.) with food available *ad libitum*. Temperature and humidity were kept at 21 ± 1°C and 50–60%, respectively. All experiments were performed during the light phase of the circadian cycle, between 09:00 and 17:00 with mice randomized across groups, in order to minimize variability related to circadian fluctuations in microglial function (Guzman-Ruiz et al., 2023).

### Elimination of microglia

C57BL/6J mice were fed a chow diet containing the CSF1R inhibitor, PLX5622 (1,200 mg PLX5622 in 1 kg chow; Plexxikon) to eliminate microglia from the brain or with control diet for 3 weeks (Szalay et al., 2016). Animals were maintained continuously on PLX5622-containing chow throughout the depletion period and during all experimental recordings to ensure stable microglial elimination and prevent repopulation.

### Whisker stimulation protocol

We performed automated whisker stimulation with a custom-designed device, using a bending actuator (#PL112-PL140; PICMA; bender connected to a piezo amplifier, #E-650 Amplifier, Physik Instrumente) at 5 Hz stimulation frequency (Csaszar et al., 2022). The actuator was positioned to stimulate the left whiskers, passively moving them both forward and backward in an alternating manner. For electrophysiology recordings, experiments were carried out under mild ketamine–medetomidine anesthesia (30 and 0.1 mg/kg, respectively, i.p.). The stimulation protocol for electrophysiology recordings consisted of 1-s-long stimulation epochs with a 100 ms on–100 ms off duty cycle, repeated 15 times in a 15 s time period, followed by a 40 s pause. The entire protocol was repeated 10 times. During control stimulation, we repeated this protocol, but the stimulator was positioned close to the whiskers without touching them. We also carried out manual whisker stimulation for comparison, using a handheld earpick, matching the stimulation frequency of the automated whisker stimulation (4–5 Hz; 15 s stimulation with 40 s interstimulus period, repeated two times). For fUS experiments, animals were briefly anesthetized with isoflurane for shaving, probe positioning, and angio3D scan acquisition. Isoflurane was then gradually replaced with medetomidine (bolus 0.1 mg/kg, i.p., followed by continuous subcutaneous infusion at 0.2 mg/kg, 7 µl/min). Under this mild medetomidine sedation, whisker stimulation was conducted: the right whiskers were manually stimulated at 4–5 Hz for 30 s, followed by 30 s of rest; this cycle was repeated 12 times.

### In vivo electrophysiology under mild anesthesia

We used custom-built implants with 8 moveable tetrode (nichrome, 12.7 µm diameter, Sandvik) electrodes (Kvitsiani et al., 2013; Hangya et al., 2015; Csaszar et al., 2022). The tetrodes were mounted on a 3D-printed skeleton, attached to a shuttle piece moved by a precision screw (stainless steel, 12 mm length, 0.6 mm outer diameter, 160 µm pitch, Easterntec) and connected to an Electrode Interface Board (Neuralynx) holding a connector (Omnetics) using gold pins (Neuralynx). They were gold plated to impedances of 30–100 kΩ measured at 1,000 Hz (NanoZ, Neuralynx) using a solution of polyethylene glycol dissolved in deionized water to 1 g/L concentration (1.125 ml) mixed with gold plating solution (0.375 ml, Neuralynx).

The tetrode microdrives were implanted via standard stereotaxic surgeries. Mice were anesthetized with a mixture of ketamine and xylazine (83 mg/kg ketamine and 17 mg/kg xylazine, dissolved in 0.9% saline, i.p.); the skin above the calvaria was shaved, disinfected (Betadine), infused by local anesthetic (lidocaine), and opened. Mice were placed in a stereotaxic frame (David Kopf Instruments). The eyes were protected by eye ointment (Corneregel, Bausch & Lomb). The skull was cleaned and leveled. A craniotomy was opened, and the implant was lowered into the right barrel cortex (anteroposterior, −1.4; mediolateral, 3.0; and dorsoventral, 0.75–2.0 mm) using the stereotaxic arm. The entry point was covered by silicon sealant (Kwik-Cast, World Precision Instruments), and the implant was secured by dental cements (MetaBond, Parkell, and dental acrylic, Lang Dental). Reference and ground wires were inserted into the contralateral parietal plate and occipital plates (30 AWG insulated copper wires). Mice received postoperative analgesics (buprenorphine, 0.1 mg/kg) and local antibiotics (neomycin). They were closely monitored after surgery and were allowed 10–14 d of recovery.

Fluorescent dye (DiI, Invitrogen LSV22885) was applied on the tip of the tetrodes before lowering for later histological localization. The electrodes were carefully positioned by moving the microdrive in small steps (20–40 µm) on the days before starting the recordings to maximize unit yield. Thus, recordings from both superficial and deep cortical layers were performed. Postmeasurement histology has also been performed to confirm electrode placement.

During recordings, a 32-channel head stage (Intan) was connected to the Omnetics connector. Data were acquired by a data acquisition system (Open Ephys), digitized at 30 kHz, synchronized with the whisker stimulator through a pulse generator (PulsePal, Sanworks). We conducted 2–3 recording sessions per mice, and tetrodes were lowered between recording sessions (40–120 μm based on the estimated electrode positions and the presence of single units) to collect neuronal activity from different dorsoventral positions. Recordings were performed under mild ketamine–medetomidine (30 and 0.1 mg/kg, i.p.) sedation.

Note that these data were previously used in Csaszar et al. (2022).

### Histology

After concluding the last recording session, mice were deeply anesthetized (83 mg/kg ketamine and 17 mg/kg xylazine, dissolved in 0.9% saline) and perfused transcardially (4% paraformaldehyde, PFA). The brains were carefully removed and postfixed overnight in 4% PFA. Fifty-micrometer-thick sections containing the tetrode tracks in the barrel cortex were cut on a vibratome (Leica), mounted on microscopy slides and positioning of the tetrodes in the barrel cortex was confirmed using a Nikon C2 confocal microscope (Nikon Instruments) by aligning images with the corresponding sections of the stereotaxic mouse brain atlas (Paxinos and Franklin, 2001) similar to Kiraly et al. (2020). Implant tip positions were determined based on the Dil marked tracks and electrical lesions performed after the recordings (Fig. S1*A*).

Microglia depletion efficiency was validated with Iba1 immunostaining. Free-floating brain sections were blocked with 5% normal donkey serum (Jackson ImmunoResearch Laboratories) and then incubated with guinea pig anti-Iba1 (Synaptic Systems, 1:500, #234004). After washing in TBS, sections were incubated with the secondary antibody: donkey anti-guinea pig A488 (Jackson ImmunoResearch Laboratories; 1:1,000; #706-546-148). After incubation, sections were washed in TBS and then mounted on glass slides using Aqua-Poly/Mount (Polysciences).

Immunofluorescence was examined using a Nikon Eclipse Ti-E inverted microscope with a 20× objective. Quantification of Iba1 immunostaining in control and microglia-depleted tissues was performed in at least three randomly selected fields of view within the cortex and the thalamus on three different coronal planes in each mouse. Data collected from every mouse brain were averaged and compared between groups.

### Spike sorting

Data analysis was carried out in MATLAB R2016a and R2016b (MathWorks). Spike sorting and classification were done blind to condition. Spikes of putative single units were sorted manually using MClust 3.5 (David Redish). Spike waveforms and autocorrelations were checked manually, and an additional effort of spike isolation was attempted if autocorrelation violations were present. If significant autocorrelation violations persisted, the spike cluster was rejected. Putative single units with appropriate cluster quality measures [isolation distance > 20 and L-ratio < 0.15 (Schmitzer-Torbert et al., 2005) were included (Hangya et al., 2015)].

### Identification of putative barrel cortex pyramidal neurons and interneurons

Putative pyramidal and interneurons were identified based on peak-to-postvalley time. First, peak-triggered average waveforms were calculated from the channel with the highest spike amplitude for each neuron (Fig. 2*A*, right). Second, postpeak valleys (local minima) were detected within a 0.6 ms time window from the peak. The distribution of the resulting peak-to-valley times exhibited a clear bimodal pattern (Fig. 2*A*, left), allowing us to categorize neurons with peak-to-postvalley time shorter than 0.19 ms as narrow-spiking (putative interneurons) and those with peak-to-postvalley time above 0.19 ms as wide-spiking neurons (putative pyramidal cells) in line with Csicsvari et al. (1999) and Vijayan et al. (2010).

### Identification of stimulus adapting and facilitating neurons

**Figure 1.**
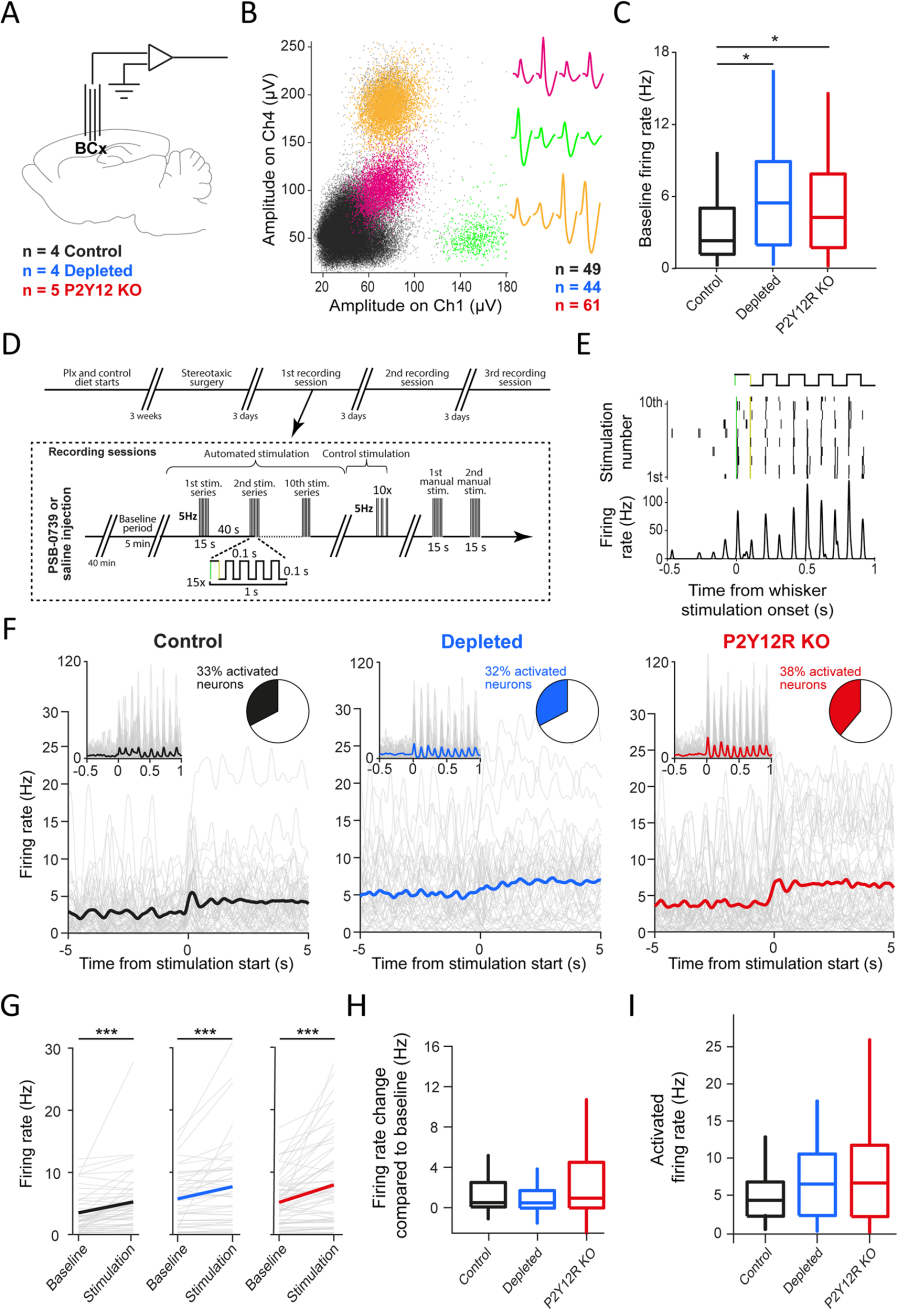
Single-unit recordings from microglia-manipulated mice reveal increased spiking activity in the barrel cortex compared with control animals. ***A***, 32-channel custom–built tetrode drives were implanted in the barrel cortex of *n* = 4 control, *n* = 4 microglia-depleted, and *n* = 5 P2Y12R KO mice. ***B***, Spikes in a 2D projection of the feature space (Ch, tetrode channel) show three single-unit examples from a control recording (left) with corresponding spike waveforms on the four tetrode channels (right). Magenta, green, and orange indicate spikes from three well-isolated clustered units, while black dots represent the remaining multiunit cluster. In total 49, 44, and 61 single-unit activities were identified in control, microglia-depleted, and P2Y12R KO mice, respectively. ***C***, The baseline firing rate of single barrel cortex units were elevated in microglia-depleted (*p* = 0.01252) and P2Y12R KO mice (*p* = 0.04992) compared with controls (two-sided Mann–Whitney *U* test; *p < 0.05). Box-and-whisker plots show median, interquartile range, and nonoutlier range. ***D***, Schematics of the whisker stimulation protocol. Whisker stimulation was applied at 5 Hz for 15 s, causing alternating passive deflections of the vibrissae, followed by a 40 s pause, repeated 10 times. This protocol was repeated without the stimulator touching the whiskers for control. Next, whiskers were manually stimulated two times for 15 s. ***E***, Example of a barrel cortex neuron activated by whisker deflections. Top, Stimulation cycles. Middle, Raster plot representing spike times aligned to whisker stimulation onset. Bottom, Peristimulus time histogram showing mean firing responses of the same neuron. ***F***, Peristimulus time histograms of all barrel cortex neurons (gray) with their average (black, control; blue, microglia-depleted; red, P2Y12R KO), aligned to stimulation onset. Left inset, Shorter timescale at higher temporal resolution shows the impact of individual stimuli. Right inset, Proportion of neurons significantly activated by stimulation start (one-sided Wilcoxon test between pre- and poststimulation firing rates, *p* < 0.05). ***G***, Stimulation-induced firing rates were increased compared with baseline in all groups (two-sided Wilcoxon signed-rank test; ***p < 0.001; control, *p* = 0.0000032; microglia-depleted, *p* = 0.00060; P2Y12R KO, *p* = 0.0000098). ***H***, The absolute change in firing rates did not differ significantly in microglia-manipulated animals compared with controls (two-sided Mann–Whitney *U* test; microglia-depleted compared with control, *p* = 0.1112; P2Y12R KO compared with control, *p* = 0.1057). ***I***, Stimulation-induced firing rates were nonsignificantly elevated in microglia-depleted and P2Y12R KO mice compared control animals (two-sided Mann–Whitney *U* test; microglia-depleted compared with control, *p* = 0.4258; P2Y12R KO compared with control, *p* = 0.4326). Box-and-whisker plots show median, interquartile range and nonoutlier range.

Adaptation of single-unit responses to repeated whisker stimulation was examined through peristimulus time histograms aligned to stimulation onset. The automated actuator moved the whiskers forward and backward within each 200 ms cycle (5 Hz), eliciting single-unit responses modulated at up to 10 Hz (Fig. 1*E*). Local response peaks separated by at least 90 ms were detected using MATLAB's findpeaks function. Spearman correlation between peak amplitude and latency from the first stimulus was calculated, where negative and positive correlations indicated adapting and facilitating neurons, respectively.

### Burst properties of barrel cortex neurons

First, autocorrelograms (ACGs) were computed with a time lag resolution of 0.5 ms to visualize temporal correlation patterns. Next, the burst index was determined: the difference between the maximum ACG value for lags of 0–10 ms and the mean ACG value for lags of 140–160 ms (chosen in such a way to best avoid the peak effects of the 5 Hz whisker stimulation) was normalized by the larger of the two values, resulting in an index ranging from −1 to 1 (Royer et al., 2012; Laszlovszky et al., 2020). For visualization (Fig. 3*B*), ACGs were smoothed with a ±5 ms wide moving average window. Bursts were defined as sequences of action potentials with interspike intervals shorter than 12 ms, similar to previous studies (E. Lee et al., 2021). The 12ms cutoff was chosen to include relatively slower bursts that are often observed under anesthesia. For each neuron, we calculated the mean intraburst spike number, the average burst duration, and the mean intraburst frequency, defined as the ratio of intraburst spike number minus 1 to burst duration.

### Spectral analysis of barrel cortex local field potentials

To explore cortical oscillatory dynamics, time–frequency spectrograms were calculated by applying wavelet transformation to the mean local field potential across channels using Morlet wavelets (Gabor, 1946). For quantitative comparison of spectral content between animal groups, Fourier spectra were computed using a fast Fourier transform algorithm (Frigo and Johnson, 1998), and the integrated power was calculated in the slow-wave (0.7–2 Hz) and upper delta (2–4 Hz) ranges of the delta band. Additionally, for each neuron, the local field potential from the corresponding tetrode was filtered in the slow-wave (0.7–2 Hz) frequency band. The filtered signals were then Hilbert-transformed to extract the instantaneous phase values of the analytical signal (Rosenblum et al., 1996; Schäfer et al., 1999). Spike phases were obtained by extracting the instantaneous phase values corresponding to the spike times. Phase locking was quantified by calculating the mean phase and the mean resultant length as the angle and magnitude of the first trigonometric moment of spike phases representing preferred phase and coupling strength, respectively [similar to Király et al. (2023)].

### In vivo electrophysiology in freely behaving mice

We performed intracranial measurements in freely behaving mice using 64-channel silicon probes (A1x64-Poly2-6mm-23s-160-H64_30 mm, NeuroNexus). The 4–5-month-old C57BL/6 mice underwent surgical implantation of polytrodes under isoflurane anesthesia; hippocampal probes were mounted on custom-made, adjustable microdrives; and the probe–microdrive assemblies were shielded by copper mesh, preventing the recordings from contamination by environmental electric noise. The mesh was covered by dental acrylic. The craniectomy was sealed by Dura-Gel (Cambridge NeuroTech) to prevent the damage of tissue and probe. Before finishing the surgery, buprenorphine (dose, 0.045 μg/g body weight) was injected subcutaneously. Recordings were started after a 1-week-long postsurgery recovery and habituation to connectorization. Recording sites spanned from the primary somatosensory cortex to the str. radiatum of the dorsal hippocampal CA1 region. Mice were observed in their home cages, and signals were acquired using an RHD2000 interface board (Intan Technologies) at a sampling rate of 20 kHz. After control recordings, mice were fed a chow diet containing the CSF1 receptor antagonist, PLX5622 (Plexxikon) for 3 weeks to eliminate microglia from the brain, and recordings were repeated, while PLX5622 diet continued. Probe positions and depletion success were evaluated after transcardial perfusion of the mice.

Spike trains were detected, clustered, and sorted using SpyKING CIRCUS (v1.1.0), Yger et al. (2018), a median filter was applied for movement artifact removal. Resulting clusters were manually curated in Phy 2.0 evaluating average spike waveforms, ACGs, amplitude distributions, and principal component projections. Further analyses were carried out in MATLAB with custom-written scripts. For spike width estimation, mean waveforms were calculated by averaging a randomly selected subset corresponding to 10% of all detected spikes per unit, sampled at 20 kHz. These waveforms were linearly upsampled to 200 kHz to improve temporal precision, and spike width was defined at the half of the mean waveform's largest amplitude. This experiment was performed specifically for Figure S1 to confirm the main findings obtained from electrophysiological recordings in anesthetized mice presented in the other figures.

### fUS imaging

fUS acquisitions were done with a 15 MHz IcoPrime-4D MultiArray probe (ICONEUS) connected to the Iconeus One fUS imaging system (ICONEUS). For each Power Doppler image, 200 frames were captured at a frame rate of 500 Hz. Each frame is a compound frame created using a set of ultrasound plane wave emissions at eight different angles (−12, −8.6, −5.1, −1.7, 1.7, 5.1, 8.6, 12°) at a 5,500 Hz pulse repetition frequency. These power Doppler images were acquired continuously at a frame rate of 0.4 s.

The fUS system detects cerebral blood volume (CBV) changes using ultrafast Doppler ultrasound (Mace et al., 2011). Since CBV is tightly coupled to neuronal activation through neurovascular coupling (Iadecola, 2017), fUS provides an indirect, high-resolution measure of neural activity and connectivity. Functional connectivity was measured noninvasively through the intact skull. The head of the animal was fixed into a stereotaxic frame directly beneath the fUS probe under 1–1.5% isoflurane anesthesia. After shaving the hair, ultrasound gel was applied to the cleaned skin. Ophthalmic gel was used to prevent eye dehydration during the experiment. The body temperature of mice was maintained at 37 ± 0.5°C using a homeothermic blanket. The heart rate and respiration rate were monitored using a Physiological Monitoring System (Harvard Apparatus).

An angio3D scanning of the brain's blood vessels was obtained, followed by aligning the scan with a mouse brain atlas using software-based registration with the 3D Allen Brain Atlas (IcoStudio 1.7 ICONEUS). Based on the atlas, the brain regions of interest were selected, and the probe was positioned accordingly. Then isoflurane anesthesia was changed to mild medetomidine sedation. After administering a bolus dose of medetomidine (Domitor, 0.1 mg/kg body weight) intraperitoneally, the isoflurane concentration was gradually reduced over 10–12 min. Then the anesthesia was maintained through a continuous subcutaneous medetomidine infusion (0.2 mg/kg body weight; 7 µl/min) during functional connectivity imaging. First, we conducted a 6 min imaging session to study resting-state functional connectivity. Then, 30 s whisker stimulation periods, each separated by a 30 s rest, were performed and repeated 12 times for a total acquisition duration of 750 s. For analysis, the 30 s stimulation periods were selected and time concatenated; the total stimulation duration was 360 s.

The preprocessing of the data was done with the IcoStudio software (ICONEUS) according to the following steps. Datasets were filtered with a high-pass filter with a cutoff frequency of 0.1 Hz. It was followed by baseline correction to remove low-frequency fluctuations by fitting a fourth-order polynomial to the data and subtracting it from the raw data, which fluctuations were caused by physiological noise. The next step was global signal regression to eliminate large-scale fluctuations, such as motion artifacts common at the whole brain, using singular value decomposition to estimate the global signal (first principal component), which was then removed from the time series of each voxel through linear regression (Fox et al., 2009; Murphy et al., 2009). The temporal Pearson's correlation coefficient (*r*) between each pair of ROIs was calculated and showed in a correlation matrix.

### Statistical analysis

Comparisons of electrophysiology data were performed using paired (Wilcoxon signed-rank test) and nonpaired (Mann–Whitney *U* test) nonparametric tests as appropriate, since normality of the data cannot be assessed statistically. Barrel cortex neurons significantly activated at the onset of whisker stimulation were identified using a one-sided Wilcoxon signed-rank test, as we specifically tested for increases in activity in response to stimulation. In other cases, two-sided tests were used. Functional connectivity data were analyzed by the GraphPad Prism 9.0 software. Data were assessed for normal distribution using the Shapiro–Wilk *W* test to determine parametric or nonparametric analysis. For comparing two groups with normal distribution, unpaired *t* test with Welch's correction was performed, and for non-normal distribution, the Mann–Whitney *U* test was used. Sample sizes were chosen based on previously published rodent in vivo imaging studies using similar experimental approaches (Wake et al., 2009; Tremblay et al., 2010; Gesuita et al., 2022; Anzibar et al., 2024) and on our own preliminary data, considering the expected variability of these in vivo imaging studies. While the analyses were performed using appropriate statistical tests as described above, some comparisons may have limited power to detect subtle effects, and this should be considered when interpreting the results.

## Results

### Microglial dysfunction is accompanied by elevated barrel cortex activity

First, we aimed to determine whether basic firing and stimulus response properties of sensory cortex neurons are altered in mice in which microglial function is compromised. Because P2Y12R shows microglia-specific expression in the brain (Butovsky et al., 2014; Fekete et al., 2018; Cserép et al., 2020), we recorded single neuron activity by using chronically implanted electrodes from the barrel cortex of anesthetized control mice, P2Y12R KO mice, or after depletion of microglia with the CSF1R inhibitor PLX5622 (Fig. S1*A*,*B*; Csaszar et al., 2022; *n* = 154 neurons; Fig. 1*A*,*B*). Baseline (prestimulus) firing rates in control mice showed a skewed distribution with a median of 2.31 Hz, consistent with earlier reports (Vijayan et al., 2010). In contrast, baseline firing rates of barrel cortex neurons were significantly elevated in both the microglia-depleted (5.46 Hz; *p* = 0.01252; Mann–Whitney *U* test) and the P2Y12R KO (4.26 Hz; *p* = 0.04992; Mann–Whitney *U* test) group (Fig. 1*C*). To assess whether microglia perturbation affects neuronal spike shape, we performed in vivo electrophysiological recordings in freely moving mice. Spike shape analysis revealed no difference in spike width between control and microglia-depleted mice, indicating that microglia depletion per se did not affect the overall pattern of neuronal spike shapes (Fig. S1*C*–*E*).

### Microglia dysfunction is associated with altered barrel cortex reactivity

Next, we tested whether sensory stimulation in anesthetized mice resulted in a firing rate increase of sensory cortical neurons (Fig. 1*D*). To this end, we delivered rhythmic mechanical stimulation of the vibrissae with a self-devised servo-based stimulator attached to the whiskers. As expected (Arabzadeh et al., 2003; Curtis and Kleinfeld, 2009; Hires et al., 2015; Petersen, 2019), whisker stimulation evoked time-locked spike responses of single barrel cortex neurons, either after retraction or protraction or both (Fig. 1*E*,*F*). We quantified these spike responses as an increase in average firing rate and found that this sensory response was strongly significant on the population level in control, microglia-depleted, and P2Y12R KO groups (*p* = 0.0000032; *p* = 0.00060; *p* = 0.0000098, respectively; Wilcoxon signed-rank test; Fig. 1*G*). This stimulus-evoked firing rate increase proved to be comparable with the control group in both depleted and P2Y12R KO mice (Mann–Whitney *U* test; *p* = 0.4258 and *p* = 0.4326, respectively; Fig. 1*H*). Thus, while microglia-depleted and P2Y12R KO mice exhibited higher baseline firing rates in the barrel cortex, responses to whisker stimuli in absolute terms remained at control levels, leading to decreased signal-to-noise ratio of sensory responses and thus potentially diminished sensory coding abilities. When we compared firing rates during stimulation, we did not observe significant differences compared with control in either microglia-manipulated groups (Fig. 1*I*). This could be because firing rate distributions showed increased variability in both microglia-manipulated groups compared with controls, potentially also reflecting noisier sensory coding (two-sample *F* test, *p* = 0.009 for depleted and *p* = 0.0034 for P2Y12R KO).

To test whether our motorized stimulation device introduced any bias in our recordings, (e.g., due to different auditory perception of the motor sound in the three groups of mice), we confirmed the above results by manually stimulating the whiskers. We found that neurons were also activated by the manual stimulation in all three mouse groups and the stimulus-evoked firing rate changes and the activated firing rates were not different among animal groups (Fig. S2*A*). Finally, we performed “stimulation control” recordings within the same recording sessions, in which we used the motorized stimulator, but it was detached from the vibrissae while maintaining its close proximity. Control stimulation did not evoke changes in the firing rate of barrel cortex neurons (Fig. S2*B*).

### Microglial dysfunction exerts its influence primarily on putative interneurons

**Figure 2.**
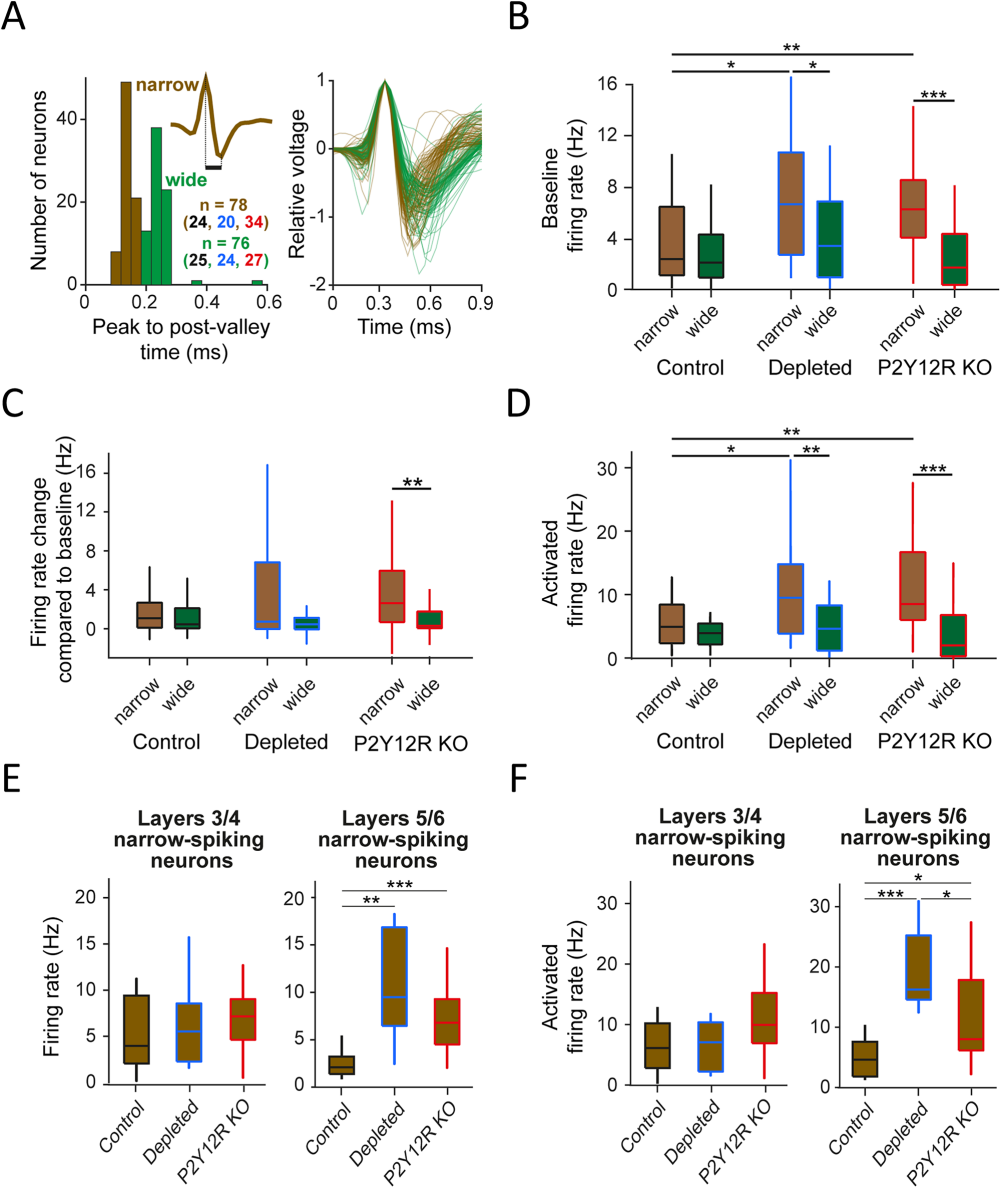
Putative interneurons account for the differences in barrel cortex activity between microglia-manipulated and control animals. ***A***, Categorization of narrow- (putative interneurons; *n* = 24; *n* = 20; *n* = 34 from control, depleted, and P2Y12R KO mice, respectively) and wide-spiking (putative pyramidal; *n* = 24; *n* = 20; *n* = 34 from control, depleted, and P2Y12R KO mice, respectively) neurons based on action potential shape. Left, Neurons with peak-to-postvalley time under 0.19 ms were considered narrow-spiking neurons, based on the bimodal distribution. Inset, Average spike shape of an example neuron demonstrating how peak-to-postvalley time (black bar) was defined. Right, Spike shapes of all recorded narrow- (brown) and wide-spiking neurons (green). ***B***, Baseline firing rates were higher in narrow-spiking neurons than in wide-spiking neurons (control, *p* = 0.5287; microglia-depleted, *p* = 0.03489; P2Y12R KO, *p* = 0.00006.) and elevated for narrow- but not for wide-spiking neurons in microglia-depleted (narrow-spiking, *p* = 0.01569; wide-spiking, *p* = 0.267) and P2Y12R KO mice (narrow-spiking, *p* = 0.00358; wide-spiking, *p* = 0.42033) compared with control animals. ***C***, Firing rate change during stimulation compared with baseline was significantly different between narrow- and wide-spiking neurons in P2Y12R KO mice (*p* = 0.00453) but not in control (*p* = 0.4902) and microglia-depleted mice (*p* = 0.1753). ***D***, Activated firing rates during stimulation were higher in narrow-spiking neurons than in wide-spiking neurons (control, *p* = 0.2113; microglia-depleted, *p* = 0.0092; P2Y12R KO, *p* = 0.00002) and significantly elevated for the narrow- but not for wide-spiking neurons in microglia-depleted (narrow-spiking, *p* = 0.0349; wide-spiking, *p* = 0.69956) and P2y12R KO mice (narrow-spiking, *p* = 0.00616; wide-spiking, *p* = 0.28675) compared with control animals. ***E***, Baseline firing rates of Layers 5/6, but not Layers 3/4, barrel cortex narrow-spiking neurons were significantly elevated in microglia-depleted (Layers 3/4, *p* = 0.5657; Layers 5/6, *p* = 0.0030) and P2Y12R KO mice (Layers 3/4, *p* = 0.3413; Layers 5/6, *p* = 0.0002) compared with controls. ***F***, Activated firing rates during stimulation were significantly higher in Layers 5/6, but not in Layers 3/4, narrow-spiking neurons of microglia-depleted (Layers 3/4, *p* = 0.8362; Layers 5/6, *p* = 0.0002) and P2Y12R KO mice (Layers 3/4, *p* = 0.1167; Layers 5/6, *p* = 0.0166), compared with control. Box-and-whisker plots show median, interquartile range and nonoutlier range. *p < 0.05; **p < 0.01; ***p < 0.001; two-sided Mann–Whitney *U* test.

Cortical neurons can be categorized as putative interneurons and putative pyramidal cells based on their action potential width (Csicsvari et al., 1999; Vijayan et al., 2010); thus, we wondered whether the increased firing rate of microglia-depleted and P2Y12R KO mice at the population level was driven by an increased firing of pyramidal neurons or interneurons or both. Our spike shape analysis revealed a bimodal distribution of peak-to-postvalley times that characterize spike width, indicating separate populations of narrow- versus wide-spiking neurons (Fig. 2*A*). These groups likely correspond to putative fast-spiking interneurons and pyramidal cells according to previous literature (Csicsvari et al., 1999; Buzsáki, 2006; Kvitsiani et al., 2013). Consistent with this, we found higher firing rates of narrow-spiking versus wide-spiking neurons (significant for the depleted and P2Y12R KO groups; Fig. 2*B*). Interestingly, we recorded a comparable number of narrow- and wide-spiking neurons despite the known dominant prevalence of pyramidal neurons over narrow-spiking interneurons, probably due to the combination of reported lower firing rates in anesthetized or sleeping versus awake animals (Erchova et al., 2002; Vijayan et al., 2010) and a sampling bias toward higher firing neurons (Henze et al., 2000). We found significantly increased baseline firing rate in narrow-spiking neurons of the depleted and P2Y12R KO mice (Fig. 2*B*; firing rate change compared with baseline significantly different between narrow- and wide-spiking barrel cortex neurons in P2Y12R KO mice; Fig. 2*C*). Similar results were obtained concerning poststimulus firing rates (Fig. 2*D*). Notably, this increased firing was largely accounted for by narrow-spiking neurons recorded from deeper cortical layers (Layers 5 and 6; Fig. 2*E*,*F*), which contain a high density of fast-spiking parvalbumin–expressing (PV^+^) interneurons (Kooijmans et al., 2020). As expected (Natan et al., 2017; Seay et al., 2020), the majority of neurons exhibited decreasing responses to repeated stimuli in control and P2Y12R KO (Fig. S3*A*). Interestingly, this adapting pattern was disrupted in neurons from Layers 3/4 of depleted mice (Fig. S3*B*), suggesting an altered inhibitory influence from somatostatin-expressing (SOM^+^) interneurons, known to regulate this adaptation in pyramidal neurons through facilitating responses to repeated stimulation (Natan et al., 2017; Seay et al., 2020; Gesuita et al., 2022). Neurons characterized by facilitating responses in our recordings also showed higher baseline firing, which was significant in microglia-depleted mice (Fig. S3*C*), whereas analysis of adaptive neurons indicated that the increased narrow-spiking activity seen in depleted and P2Y12R KO mice persisted even without the facilitating neurons (Fig. S3*D*). These results suggest that compromising brain microglia, by either chronic depletion or interference with their P2Y12R, leads to an increase in interneuron excitability in the cortex that may impair reliable sensory representations.

**Figure 3.**
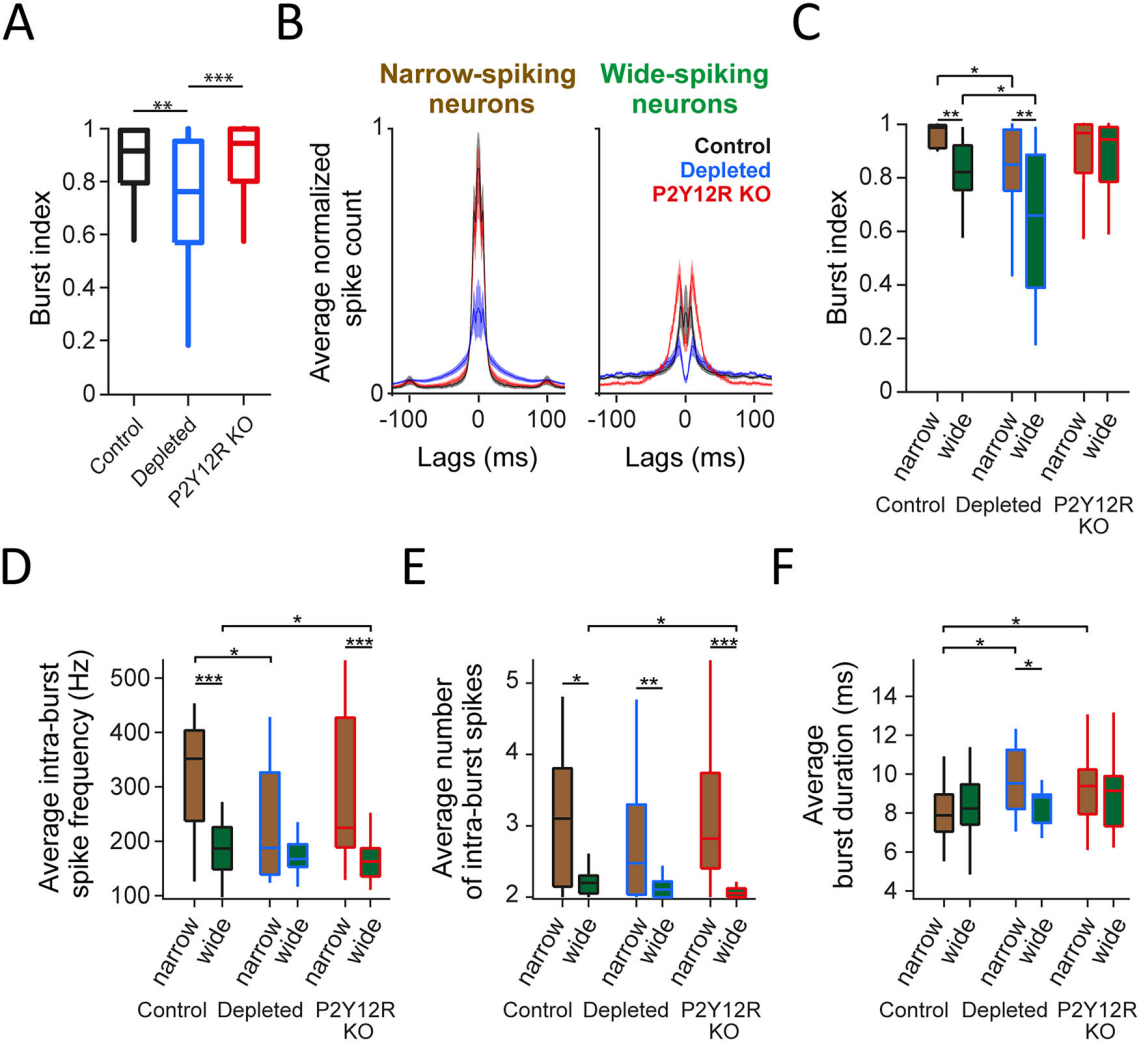
Reduced burstiness in the barrel cortex of microglia-depleted animals. ***A***, Burst index distributions of all neurons across animal groups showed reduced bursting in microglia-depleted animals compared with both control (*p* = 0.00534) and P2Y12R KO mice (*p* = 0.00005). ***B***, Average ACGs of narrow- and wide-spiking neurons in each animal group. ***C–F***, Comparison of burst index (***C***), average intraburst frequency (***D***), average number of intraburst spikes (***E***), and average burst duration (***F***) between narrow- and wide-spiking neurons within and across animal groups. In microglia-depleted animals, burstiness was reduced in both narrow- (*p* = 0.02837) and wide-spiking neurons (*p* = 0.03001). Intraburst spike frequency was significantly reduced in narrow-spiking neurons of microglia-depleted (*p* = 0.0179) and wide-spiking neurons of P2Y12R KO mice (*p* = 0.0459). The intraburst spike number was decreased in wide-spiking neurons of P2Y12R KO mice (*p* = 0.0140). Burst duration was significantly increased in narrow-spiking neurons of both depleted (*p* = 0.0123) and P2Y12R KO (*p* = 0.0356) mice. Box-and-whisker plots show median, interquartile range and nonoutlier range. *p < 0.05; **p < 0.01; ***p < 0.001; two-sided Mann–Whitney *U* test.

Burst firing in sensory cortices is mediated by a combination of intrinsic properties and specific inputs, and bursts are thought to have distinct coding properties and functions (Otto et al., 1991; Reinagel et al., 1999; Kepecs et al., 2002; Kepecs and Lisman, 2003; Laszlovszky et al., 2020; Hegedus et al., 2021). Therefore, we tested whether burst firing of barrel cortex neurons was altered when microglia function was compromised. We found that burstiness was decreased in microglia-depleted mice, while a normal level of burstiness was maintained in P2Y12R KO mice (control vs microglia-depleted; *p* = 0.00534; two-sided Mann–Whitney *U* test; Fig. 3*A*). Burst firing occurs both in interneurons and pyramidal cells (Zhu and Connors, 1999; Swadlow, 2003; Jacob et al., 2012); thus, we next tested narrow- and wide-spiking neurons separately (Fig. 3*B*). Both narrow-spiking and wide-spiking neurons of microglia-depleted mice showed decreased burst firing (narrow-spiking, *p* = 0.02837; wide-spiking, *p* = 0.03001; two-sided Mann–Whitney *U* test; Fig. 3*C*). Although P2Y12R KO mice did not show a significant reduction in burst firing, slowing of their bursts was indicated by an analysis of intraburst frequency (Fig. 3*D*; significantly lower for wide-spiking neurons, *p* = 0.0459), fewer spikes per burst (Fig. 3*E*; significantly lower for wide-spiking neurons, *p* = 0.0140), and burst duration (Fig. 3*F*; significantly higher for narrow-spiking neurons, *p* = 0.0356; two-sided Mann–Whitney *U* tests), in accordance with the autocorrelation shapes in Figure 3*B*. These results suggest that sensory stimulus coding may be impaired when brain microglia are absent.

### Elevated barrel cortex slow oscillations and hypersynchrony after microglia depletion

**Figure 4.**
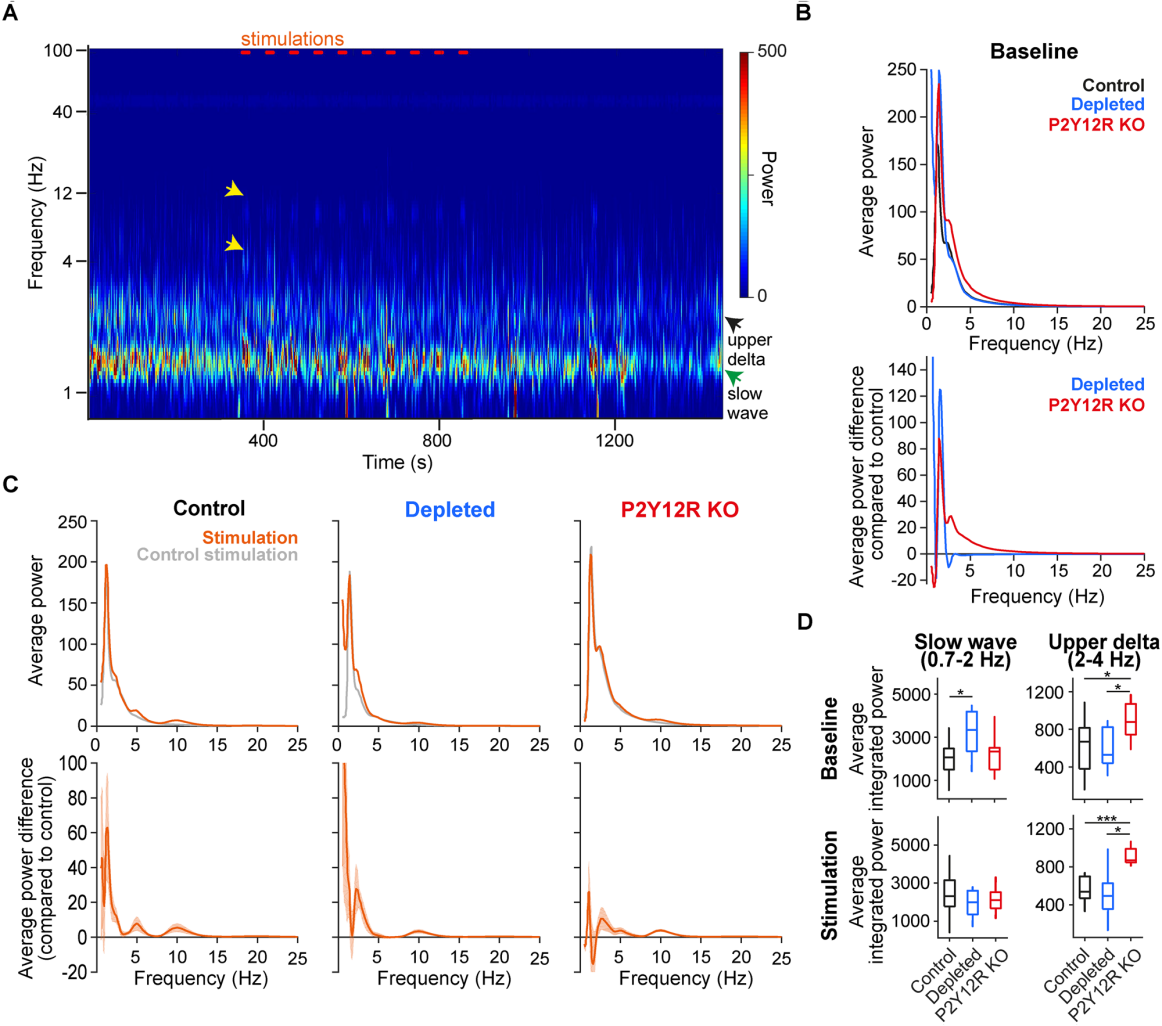
Increased barrel cortex delta activity in microglia-manipulated mice. ***A***, Wavelet power spectrogram of an example session from an anesthetized control mouse, showing two distinct components of delta activity (slow wave, 0.7–2 Hz; upper delta band, 2–4 Hz). Stimulation periods (marked by orange lines) were followed by increased 5 and 10 Hz activity (yellow arrow) reflecting sensory stimulation rate. ***B***, Top, Average Fourier power spectral density measured during stimulation and control stimulation in control, microglia-depleted, and P2Y12R KO mice. Bottom, Average difference in spectral power between stimulation and control stimulation periods. ***C***, Top, Average baseline spectral power spectral density in each animal group. Bottom, Average spectral power difference between microglia-manipulated and control mice. ***D***, Integrated spectral power in the slow-wave (0.7–2 Hz) and upper delta (2–4 Hz) ranges of the delta band during baseline and stimulation periods compared across animal groups (slow-wave power during baseline compared with control, depleted, *p* = 0.035; upper delta power during baseline compared with control, depleted compared with control, *p* = 0.0438; P2Y12R KO, *p* = 0.0135; upper delta power during stimulation, depleted compared with control, *p* = 0.00046; P2Y12R KO compared with control, *p* = 0.0275). Box-and-whisker plots show median, interquartile range and nonoutlier range. *p < 0.05; **p < 0.01; ***p < 0.001; two-sided Mann–Whitney *U* tests.

Population level activity in the barrel cortex of anesthetized mice is dominated by slow (delta band) oscillations reflecting sleep-like intracortical and thalamocortical synchronization processes (Crunelli et al., 2002; Destexhe and Sejnowski, 2003; Neske, 2015). This can be interrupted by sensory stimulation, which we confirmed by demonstrating that 10 Hz whisker stimulation introduced a visible 10 Hz component in the barrel cortex spectrograms in all three groups of mice (Fig. 4*A*,*B*). We investigated whether microglia dysfunction affected delta band oscillatory synchrony. We found that lower delta band (0.7–2 Hz) spectral power was significantly higher in microglia-depleted mice compared with control animals (Fig. 4*C*,*D*; Mann–Whitney *U* test, *p* = 0.035), whereas P2Y12R KO mice showed a discrete spectral increase in the upper range of the delta band (2–4 Hz; *p* = 0.0438), especially during whisker stimulation (Fig. 4*D*; *p* = 0.00046).

**Figure 5.**
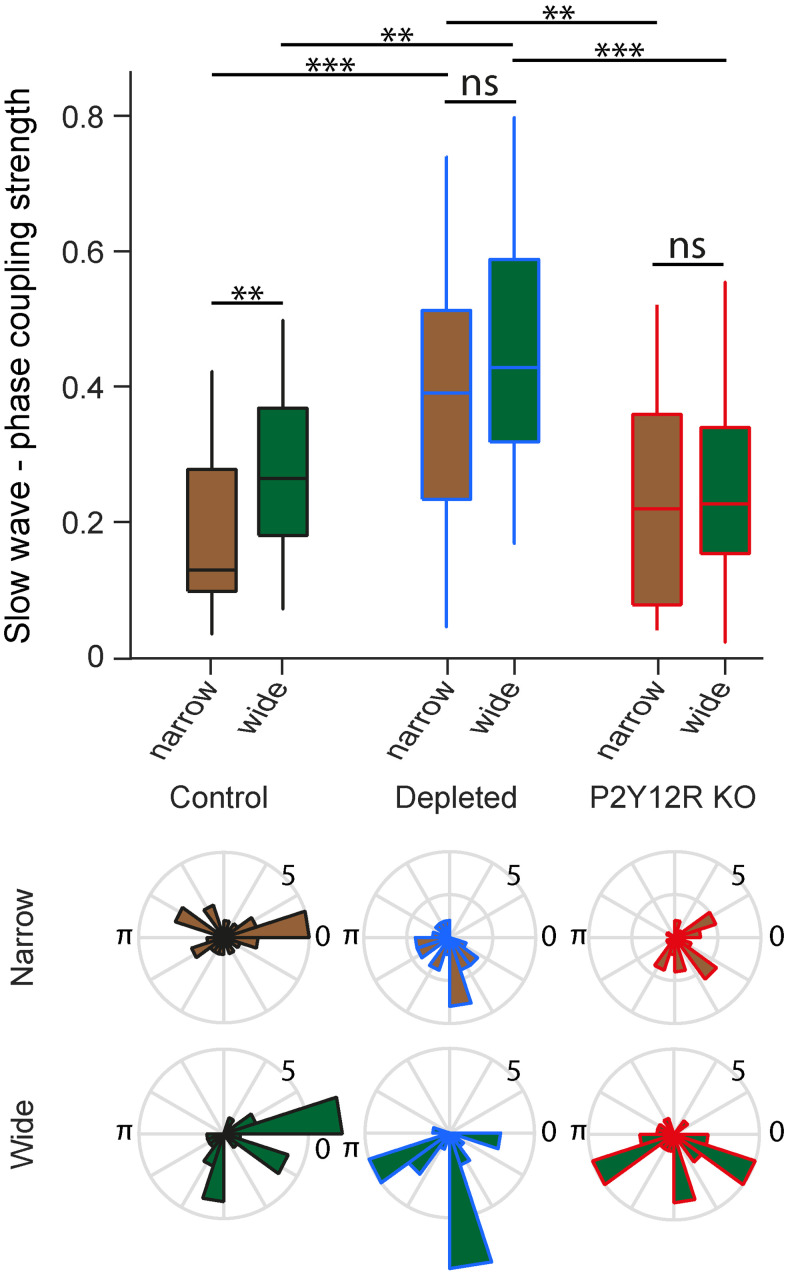
Increased slow-wave coupling in microglia-depleted but not in P2Y12R KO animals. Top, Phase coupling strength distribution of neurons to slow waves shows increased coupling strength in microglia-depleted mice compared with control and P2Y12R KO mice for both narrow- (microglia-depleted vs control, *p* = 0.0003; microglia-depleted vs P2Y12R KO, *p* = 0.0056) and wide-spiking neurons (microglia-depleted vs control, *p* = 0.0021; microglia-depleted vs P2Y12R KO, *p* = 0.0001; two-sided Mann–Whitney *U* tests). While wide-spiking neurons were more strongly coupled than narrow-spiking neurons in control mice (*p* = 0.0091), this difference was not significant in microglia-depleted (*p* = 0.3398) and P2Y12R KO (*p* = 0.6474) mice. **p < 0.01; ***p < 0.001, ns, not significant. Box-and-whisker plots show median, interquartile range, and nonoutlier range. Bottom, Preferred slow-wave (0.7–2 Hz) phase histogram of barrel cortex narrow- and wide-spiking neurons in control, microglia-depleted, and P2Y12R KO mice.

Finally, we tested local delta band synchrony in the barrel cortex by calculating phase coupling strength of neurons to local delta band oscillations. We found increased phase coupling to lower delta band slow oscillations in microglia-depleted mice in both narrow- and wide-spiking neurons, while phase coupling strength was comparable with the controls in microglia-impaired (P2Y12R KO) animals (Fig. 5). Furthermore, the stronger phase locking of wide-spiking cells compared with narrow-spiking cells observed in control animals disappeared in both microglia-manipulated groups. These results suggest delta band hypersynchrony when microglial function is compromised.

### Microglial dysfunction disrupts thalamocortical functional connectivity during somatosensory stimulation

**Figure 6.**
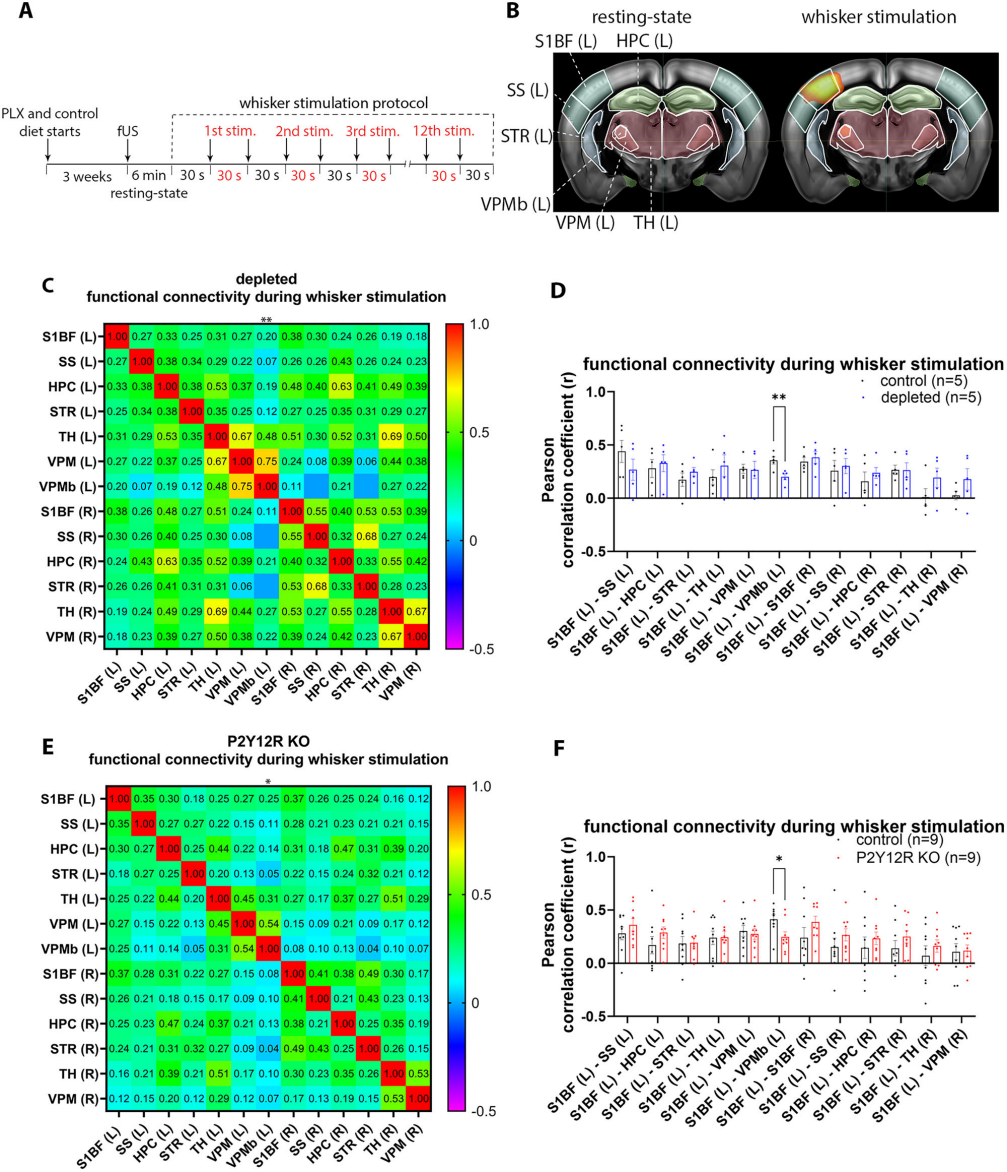
Microglia contribute to functional connectivity changes during somatosensory stimulation. ***A***, Outline of the experimental protocol. Functional connectivity was measured using fUS during resting-state and right whisker stimulation. ***B***, Representative images show the ROIs (white outlines). ***C***, Mean Pearson's correlation matrix (*n* = 5 depleted mice) during whisker stimulation. ***D***, The graph shows Pearson's correlations for pairs of ROIs. In the absence of microglia, the correlation between S1BF (L) and VPMb (L) was reduced in response to whisker stimulation compared with controls (**p < 0.01; *p* = 0.0078, unpaired *t* test with Welch's correction; *n* = 5 control and *n* = 5 microglia-depleted mice). Data are presented as mean ± SEM. ***E***, Mean Pearson's correlation matrix obtained in *n* = 9 P2Y12R KO mice during whisker stimulation. ***F***, The graph shows decreased connectivity between the S1BF (L) and VPMb (L) in P2Y12R KO mice compared with controls during whisker stimulation (*p < 0.05; *p* = 0.0197, unpaired *t* test with Welch's correction; *n* = 9 control and *n* = 9 P2Y12R KO mice). Data are presented as mean ± SEM (Tables S1 and S2). S1BF, primary somatosensory area, barrel field; SS, supplemental somatosensory area; HPC, hippocampal region; STR, striatum; TH, thalamus; VPM, ventral posteromedial nucleus of the thalamus; VPMb, ventral posteriormedial nucleus of the thalamus barreloid; (R), right; (L), left.

We showed that microglia dysfunction leads to increased slow oscillatory (delta band) synchrony in the barrel cortex reflected in both increased delta band spectral power and stronger phase locking to slow oscillatory components of individual barrel cortex neurons. This could either be due to local hypersynchrony in cortical circuits or increased thalamocortical activity that enhanced thalamocortical slow oscillatory loops (Crunelli et al., 2015). To disentangle these two possibilities, we tested whether the absence of microglia altered functional connectivity at rest and during sensory stimulation by combining our whisker stimulation protocol with fUS measurements. As fUS measures CBV changes, the observed connectivity patterns should be interpreted as reflecting underlying neuronal activity indirectly via neurovascular coupling. After microglia depletion, a 6 min resting-state epoch was recorded, followed by repeated stimulation of the right whiskers under mild medetomidine sedation (30 s ON periods followed by 30 s OFF periods, repeated 12 times; Fig. 6*A*).

We analyzed functional connectivity in the coronal plane 1.6 mm posterior to the bregma, which included the barrel cortex (S1BF) and several relevant anatomically connected sites, including the supplemental somatosensory area (SS), the hippocampus (HPC), the striatum (STR), the thalamus (TH), and the ventral posteromedial nucleus of the thalamus (VPM; Fig. 6*B*). Among these regions, CBF increases in response to whisker stimulation were observed specifically in the barrel cortex (S1BF) and the VPM (Fig. 6*B*).

No significant changes in resting-state functional connectivity were observed (Fig. S4*A*,*B*). However, during whisker stimulation, we found reduced connectivity between the contralateral barrel cortex (left S1BF) and the ventral posteromedial nucleus of the thalamus barreloid region (left VPMb) in microglia-depleted mice compared with controls (*p* < 0.01; *p* = 0.0078; unpaired *t* test with Welch's correction; Fig. 6*C*,*D*; Fig. S4*C*). Correlations between left VPMb and other ROIs did not show significant changes in the absence of microglia (Fig. S4*D*).

As P2Y12R is exclusively expressed by microglia in the brain (Butovsky et al., 2014), we investigated whether P2Y12R contributes to functional connectivity changes. We did not see significant differences in resting-state functional connectivity in the absence of P2Y12R (Fig. S4*E,F*). However, we observed a marked reduction in functional connectivity between the left S1BF and left VPMb in P2Y12R KO mice (*p* < 0.05; *p* = 0.0197, unpaired *t* test with Welch's correction; Fig. 6*E,F* and Fig. S4*G*), similar to the reduction seen in microglia-depleted mice. Analysis of correlations between VPMb and other investigated brain regions did not reveal significant alterations in P2Y12R KO mice (Fig. S4*H*). Thus, our observations suggest that microglia-mediated actions are important for maintaining normal functional connectivity in the thalamocortical network during somatosensory stimulation. We also propose that the observed cortical hypersynchrony was likely due to changes in local cortical processing as opposed to increased thalamocortical inputs.

## Discussion

Here, we reveal the modulatory role of microglia in cortical excitatory and inhibitory circuits and in functional thalamocortical connectivity. Our results provide novel mechanistic insight into complex network-level processes in which microglial actions are increasingly recognized, with particular relevance to microglia–neuron interactions during sensory processing.

Microglia depletion has been shown to exert region-specific effects on excitatory synaptic transmission, diminishing it in the motor cortex and hippocampus while enhancing it in the visual cortex and striatum (Parkhurst et al., 2013; Badimon et al., 2020; Liu et al., 2021b; Corsi et al., 2022). Modulation of neuronal activity by microglia also involves interactions with neuronal somata at somatic purinergic junctions or perisomatic GABAergic terminals via microglial P2Y12R and mediators, such as adenosine or PGE2 (Szalay et al., 2016; Badimon et al., 2020; Klawonn et al., 2021; Cserep et al., 2022; Haruwaka et al., 2024). Adding to these findings, increased baseline and poststimulus firing rates observed in narrow-spiking interneuron populations in the present study after microglial depletion or in the absence of P2Y12R strongly suggest that microglia specifically modulate interneuron excitability. We expect that the narrow-spiking group we isolated in the barrel cortex largely overlaps with PV-expressing interneurons, based on earlier studies (Csicsvari et al., 1999; Vijayan et al., 2010; Kvitsiani et al., 2013). We observed a surprisingly large population of narrow-spiking neurons (Beaulieu, 1993; Harris and Shepherd, 2015), which could be due to the known sampling bias in favor of higher firing neurons in extracellular recordings (Henze et al., 2000), combined with an inclusion of deep layer recordings where PV-expressing interneurons are highly represented (Kooijmans et al., 2020). Our findings are in accordance with a recent study that demonstrated higher in vitro activity and in vivo calcium levels of visual cortical PV-expressing interneurons after microglia depletion (Liu et al., 2021b). Neurons with facilitating responses to 10 Hz stimulation, possibly corresponding to SOM^+^ interneurons (Gesuita et al., 2022), showed alterations similar to those of narrow-spiking neurons. By fine-tuning inhibitory circuits, microglia could shift excitatory/inhibitory balance in cortical networks (Avermann et al., 2012), ultimately affecting sensory representations and other processes**.** Supporting this, the lack of normal microglial function may impair sensory processing as suggested by the finding that increased fast-spiking interneuron activity impairs sparse coding in the barrel cortex, necessary for sensory discrimination (Petersen, 2007; Avermann et al., 2012; Petersen, 2019; Staiger and Petersen, 2021). Increased firing rate variability in microglia-depleted mice may interfere with reliable stimulus representations and thus impair stimulus discrimination (Fiser et al., 2010; Haroush and Marom, 2019). Importantly, the role of microglia may vary across brain regions (Brown et al., 2024), calling for further investigation.

Altered excitatory/inhibitory balance may affect burst firing, thought to represent specialized coding mechanisms, whereby single spike and burst coding can dissociate within single neurons, leading to multiplexed information transfer (Kepecs et al., 2002; Kepecs and Lisman, 2003; Laszlovszky et al., 2020; Hegedus et al., 2021). In the neocortex, burst firing occurs both in fast-spiking interneurons and pyramidal cells (Franceschetti et al., 1995; Kawaguchi et al., 2019; Leleo and Segev, 2021). We found that microglia depletion led to decreased bursting of both types of neurons in the barrel cortex. Since bursts might signal salient sensory stimuli or those directly relevant to motor processing (Zeldenrust et al., 2018), decreased burst firing in microglia-depleted mice may lead to impaired processing of behaviorally relevant stimulus features. Although P2Y12R KO mice did not show a significant deficit in burst firing, slowing of their bursts suggested impaired intraburst dynamics.

Changes in burst firing may influence network synchronization, which occurs at different frequencies representing distinct aspects of information processing (Buzsáki, 2006; Maris et al., 2016). Microglia depletion increased pairwise synchrony of calcium events in the striatum (Badimon et al., 2020), but microglia ablation decreased synchrony in the primary motor cortex (Akiyoshi et al., 2018). Microglia depletion led to a decrease of hippocampal sharp-wave ripples in acute slices and in vivo (Berki et al., 2024). Forty hertz stimulation had beneficial effects on microglia function and amyloid clearance (Iaccarino et al., 2016), suggesting a bidirectional interaction between cortical gamma oscillations and microglia function (Çaliskan et al., 2022). A similar complex interplay was hypothesized between thalamocortical delta oscillations and microglia (Steffens et al., 2024). Here we found that microglia depletion and P2Y12R loss resulted in increased delta oscillations, suggesting hypersynchrony in the low-frequency bands if microglia function is compromised. Interestingly, while microglia depletion only affected the lower delta band often linked to slow-wave sleep, P2Y12R KO showed hypersynchrony in the upper delta band, implicated in both sleep and rest (Sachdev et al., 2015; Halgren et al., 2018; Gunasekaran et al., 2023). Interference with delta band rhythms in microglia-compromised mice may impair the integration of whisker touch with temporally correlated information from other senses (Ranade et al., 2013).

To investigate whether these cellular-level changes are associated with network-level alterations, we examined the influence of microglia on functional connectivity during somatosensory stimulation using fUS to measure activity-dependent changes in CBV. Alterations in functional connectivity between the barrel cortex and VPMb during whisker stimulation in the absence of microglia or microglial P2Y12R suggest that microglia play a critical role in modulating thalamocortical networks. These findings likely reflect a combination of altered local responsiveness and broader network effects rather than a complete decoupling of sensory processing. We therefore emphasize that the CBV-based fUS signals provide an indirect readout of network-level effects, which may be shaped by inhibitory interneurons including PV cells (Nunez-Elizalde et al., 2022). Future studies combining direct interneuron recordings or optogenetic manipulation with fUS could clarify the specific contribution of PV neurons to these hemodynamic signals. Consistent with our findings, microglia or microglial CX3CR1 signaling was shown to regulate the maturation of thalamocortical synapses in the somatosensory cortex (Hoshiko et al., 2012) and increase local excitatory and inhibitory circuit connectivity to excitatory neurons in the visual cortex, highlighting the involvement of microglia in cortical development and plasticity (Liu et al., 2021a).

In addition, recent work showed that microglial depletion during development impairs sensory-evoked cortical responses (Gesuita et al., 2022), underscoring differences between developmental and adult roles of microglia. While early depletion may affect inhibitory circuit maturation, including SOM interneurons, our results suggest that in the adult brain, microglia continue to modulate sensory processing through dynamic modulation of inhibitory circuits. In line with this, we found altered adaptability in barrel cortex Layer 3/4 neurons in microglia-depleted mice, but not in P2Y12R KO animals, suggesting a role of microglia in maintaining cortical sensory adaptation through mechanisms beyond P2Y12R signaling. Although our results suggest involvement of both SOM^+^ and PV^+^ interneurons, differences in stimulation protocols may affect the precise response patterns and are important to consider when comparing across studies.

The largely overlapping effects of microglia depletion and P2Y12R deficiency strongly argue for the pivotal role of microglia in cortical network activity and functional connectivity changes after somatosensory stimulation via prominent effects on inhibitory neuronal populations; nonetheless, several methodological considerations should be noted. Pharmacological microglia depletion by using CSF1R inhibitors provides a broad approach which has been suggested to trigger compensatory mechanisms including vascular adaptation if performed for several weeks (Yang et al., 2020). However, microglia depletion does not have a detrimental effect on neurons, astrocytes, pericytes, and other brain cells, and only mild side effects on peripheral myeloid cells and other immune cells have been identified (Elmore et al., 2014; Cserép et al., 2020; Okojie et al., 2023). Nevertheless, developmental adaptations in germline KO models cannot be fully excluded. While we investigated exclusively male mice, both microglial actions and disorders with altered E/I balance involve sex-specific mechanisms (Villa et al., 2019), warranting further studies. The observed alterations in baseline firing, burst activity, and thalamocortical connectivity provide mechanistic insight into how microglia modulate cortical sensory processing, which should be extended by addressing the behavioral consequences of these cellular and network-level changes in future reports.

We and others have shown that microglia play instrumental roles in neurovascular coupling via P2Y12R (Csaszar et al., 2022; Fu et al., 2025), which could involve actions via interneurons. We note that a contribution of basal firing rate increase of specific interneuron classes to altered blood flow regulation cannot be excluded, supported by reduced blood flow upon increased PV-interneuron activity (J. H. Lee et al., 2021). Thus, by increasing the firing rate of interneurons, microglia could influence complex neurovascular processes with both direct and indirect impact on local neuronal circuits or larger networks.

Microglial dysfunction is known to lead to pathological hyperexcitability and hypersynchrony, which may underline network vulnerabilities in various neurological and neurodegenerative disorders. Hyperexcitability and hypersynchrony within cortical networks are hallmarks of conditions such as epilepsy, obsessive–compulsive disorder, and other neuropsychiatric disorders (Wan et al., 2020; Merlini et al., 2021). Thus, interventions modulating microglia and neuronal excitability may have therapeutic benefits in broad neurological conditions.
